# Is accreditation in medical education in Korea an opportunity or a burden?

**DOI:** 10.3352/jeehp.2020.17.31

**Published:** 2020-10-21

**Authors:** Hanna Jung, Woo Taek Jeon, Shinki An

**Affiliations:** Department of Medical Education, Yonsei University College of Medicine, Seoul, Korea; Hallym University, Korea

**Keywords:** Accreditation, Diagnostic self evaluation, Medical education, Republic of Korea, Self-assessment

## Abstract

The accreditation process is both an opportunity and a burden for medical schools in Korea. The line that separates the two is based on how medical schools recognize and utilize the accreditation process. In other words, accreditation is a burden for medical schools if they view the accreditation process as merely a formal procedure or a means to maintain accreditation status for medical education. However, if medical schools acknowledge the positive value of the accreditation process, accreditation can be both an opportunity and a tool for developing medical education. The accreditation process has educational value by catalyzing improvements in the quality, equity, and efficiency of medical education and by increasing the available options. For the accreditation process to contribute to medical education development, accrediting agencies and medical schools must first be recognized as partners of an educational alliance working together towards common goals. Secondly, clear guidelines on accreditation standards should be periodically reviewed and shared. Finally, a formative self-evaluation process must be introduced for institutions to utilize the accreditation process as an opportunity to develop medical education. This evaluation system could be developed through collaboration among medical schools, academic societies for medical education, and the accrediting authority.

## Introduction

### Background/rationale

The administrative burden faced by medical schools in Korea has increased as evaluations of medical schools have evolved into an accreditation process. Simultaneously, however, accreditation is an excellent opportunity since it serves as a “gold standard” for improving the quality of medical education [1]. Many studies have cited accreditation as an opportunity to reform the curriculum and strengthen educational programs [2-9].

The Flexner report, the current prototype of medical education accreditation, was done by Abraham Flexner in the early 20th century to record and evaluate medical schools in North America [10]. Flexner [10] regarded the educational model of Johns Hopkins School of Medicine as the standard model for evaluating medical schools and other related institutions. The report catalyzed major improvements in medical education [11]. After the report, underqualified medical schools quickly began to close or merge. As a result, between 1910 and 1922, the number of medical schools in the United States decreased from 131 to 81 [12]. Flexner [10] proposed the “2 plus 2” medical education model, involving 2 years of preclinical basic science education and 2 years of clinical training, and placed a greater emphasis on the curriculum and the admission process. In this way, the Flexner report became a significant turning point in medical education, not just in the United States and Canada, but also globally [13].

Medical school evaluation, which traces its roots to the 20th-century United States, is consistent with the current process of medical education accreditation. It is essential to provide external feedback to improve performance because relying only on self-evaluation may be inaccurate [14]. However, feedback through medical education accreditation is meaningful only when the accreditation body and medical schools trust each other. Medical schools (as medical education providers) and accreditation agencies (as evaluators and feedback providers) form an “educational alliance.” This concept originated from psychotherapy, where patients and therapists form a therapeutic alliance [15]. In such an alliance, it is not sufficient that the therapist merely provides feedback; instead, the feedback must be given within a relationship of trust with the patient. Likewise, the feedback from the accreditation body must be in the context of trust. Medical schools need to listen to the feedback and view it as an opportunity to improve. Likewise, the accreditation body should also listen to medical schools’ feedback, creating a mutually beneficial interaction [15].

Due to a lack of sufficient human resources to prepare for accreditation, medical school administrators may face various difficulties. Regular visits for accreditation may be regarded as burdens, rather than opportunities. Furthermore, medical educators may experience accreditation fatigue or accreditation burnout and may perceive accreditation negatively [2]. Moreover, the accreditation of medical education has become mandatory worldwide, including in Korea; as such, it has become a “must-have” option, not just a desirable option, even if it is not burdensome [3-5,7,9,13,16]. Medical schools can make more real changes if they think of accreditation as an opportunity to implement innovations or as a tool for reorganizing the curriculum.

### Objectives

This review aims to help medical schools to take full advantage of the accreditation process as an educational development opportunity. First, the meaning and goals of accreditation are explained. Second, we introduce accreditation standards and elements, and present a comparison between Korea’s model of the accreditation process and the models of other countries. Third, we discuss how the pedagogical value of education has been realized in the medical education field. Lastly, based on these discussions, we propose directions for the accreditation body and medical schools to improve medical education quality.

## Meaning and goals of medical school accreditation

Medical education accreditation is divided into evaluation, which applies to the entire medical education program, and accreditation, which involves an assessment of medical schools’ educational qualifications [17]. Many countries use accreditation as a regulatory mechanism to improve medical education [7]. However, policymakers have generally not used the corresponding evidence when attempting to make reforms, or have made incremental changes based on ideas that selectively cited the relevant evidence [18]. The results of accreditation can contribute to making medical school-related policy decisions through a rational process. In this respect, accreditation can be a powerful driving force and catalyst for medical education reform [6,19].

Another aspect of accreditation is that it serves as a safeguard between education providers and consumers, “Thus, it is expected that accreditation standards will foster the medical programs that prepare graduates to deal with new knowledge and become lifelong learners (World Health Organization, 2005)” [13]. Being accredited to provide a medical education program reflects a guarantee that the program competently manages education and training [17]. In light of the demand for medical education, accreditation can also help protect society from the impact of poor-quality medical education programs; furthermore, social trust in medical schools and physicians can be secured by confirming physicians’ essential competency [13,17]. Therefore, medical school accreditation can serve as a tool to increase medical expertise and to promote communication and interactions with society. Medical education accreditation has the ultimate goal of improving the health of the community [6,13,20]. The following objectives can be pursued to achieve the ultimate goal of accreditation [2,13]:

- To ensure the quality of educational programs
- To encourage reforms in medical school
- To enhance the public’s and stakeholders’ trust in medical schools
- To promote the international recognition of medical schools
- To provide evidence for correlations between programs and the graduates’ competency

## Components of accreditation

The key elements of accreditation are legislative establishment of the accreditation body, precise setting and presentation of accreditation standards, and effective certification procedures.

### Legislative establishment of certification bodies

**Fig. 1.**
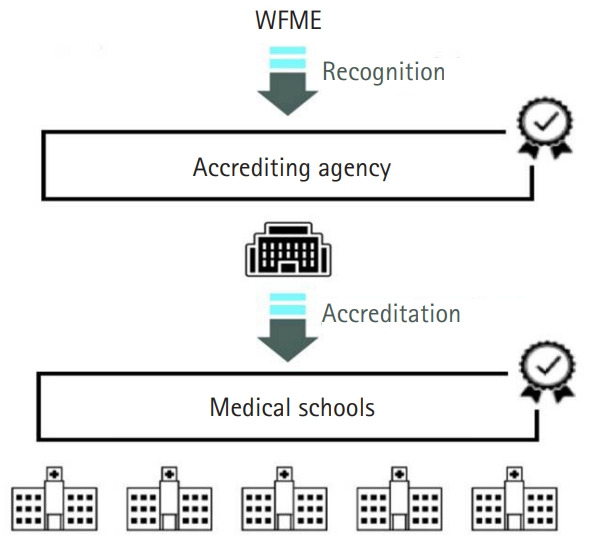
The relationship between the WFME, accrediting agencies, and medical schools. WFME, World Federation for Medical Education.

The accreditation body must be legislated as a public organization considering the impact of accreditation on medical education and public health. The types of publicly recognized accreditation bodies vary by country. In many countries, government agencies such as the Ministry of Health and Welfare and the Ministry of Education can accredit educational institutions [1]. There are also cases where the government approves independent institutions as accreditation bodies, such as the General Medical Council (GMC) in the United Kingdom, the Liaison Committee on Medical Education (LCME) in the United States, and the Korean Institute for Medical Education and Evaluation (KIMEE) in Korea. In such cases, the authority of the certification body is determined by government-approved laws [1,21]. The World Federation for Medical Education (WFME) conducts a recognition program for each country’s accreditation body (Fig. 1). The WFME’s recognition of accreditation bodies in various countries means that the quality of the medical education programs accredited by each accreditation body can be guaranteed externally [22]. As of December 2019, a total of 20 accreditation agencies were recognized by the WFME. The KIMEE was the 1st such agency recognized by the WFME in Asia and the 4th in the world. Its recognition period is 10 years, lasting from 2016 to 2026.

### Clear establishment and presentation of accreditation standards

**Fig. 2.**
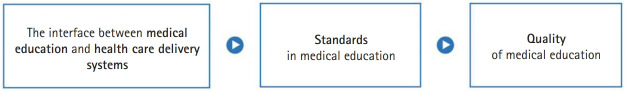
Standards for accreditation in medical education.

Since accreditation standards are influenced by the relationship between medical education and the medical delivery system, and have a direct impact on medical education quality, accreditation standards should be clearly established (Fig. 2). As physicians’ movement between countries increased, and the globalization of medicine occurred, interest in international accreditation standards for medical education has grown [23]. The WFME first published a report on international standards of medical education in 1998 [24]. In 2005, it released guidelines for basic medical education standards, including medical education curriculum, competency building, and evaluation at the regional and national levels [17]. The WFME considered the following factors in the process of developing international accreditation standards [25].

- The standards should be the driving force to review and change medical education through self-assessment by institutions.
- The standards should consider differences in medical education among countries because countries differ in culture, traditions, socio-economic potential, health, disease scope, and health care delivery system.
- The standards should not dictate education content, degrade the quality of education, ban certain educational methodologies, rank schools, or be used politically.
- The standards should emphasize the universality of the scientific foundation of medicine. Medical education aims to nurture physicians who can care for healthy, sick, disabled, or injured citizens.
- The standards can serve to build national or international medical education programs.

Establishing an international accreditation standard can improve the quality of medical education and is necessary for evaluating medical education at the national level. However, international accreditation standards may potentially harm medical schools’ autonomy through strict regulations, make medical schools focus on only the bare minimum requirements, and lead schools to merely adhere to the accreditation standards instead of attempting reforms. As international standards come with both advantages and disadvantages, it is essential to create a reliable tool to ensure medical education quality while balancing these 2 sides [23]. Furthermore, it is necessary to construct accreditation standards according to national and regional cultural factors, socioeconomic factors, and medical delivery systems [13].

### Establishing effective accreditation procedures

**Fig. 3.**
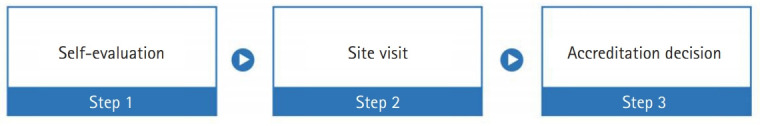
A typical medical education accreditation process.

The accreditation procedure differs depending on the accreditation body, but the process can be generally classified into 3 steps [3,13]. The first step is a self-evaluation, the next step is a site visit, and the final step is an accreditation decision by the accreditation body (Fig. 3). At the self-evaluation step, each medical school analyzes its own situation according to a predetermined accreditation standard. The result of this step is of vital importance because it is the basis of the accreditation process [3,13]. A group of external reviewers then visits the school and checks the content described in the self-evaluation report on site, which is the second step of the site visit. According to the results of the site visit, the accreditation body decides and announces the medical school's accreditation level as the final accreditation step. The specific results may vary depending on the accreditation body, but are generally classified into full, conditional, or no accreditation. The accreditation period is typically between 4 and 10 years.

**Fig. 4.**
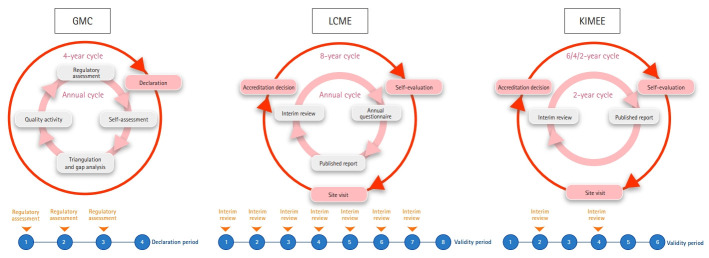
Quality assurance process of GMC, LCME, and KIMEE. Adopted from General Medical Council. Overview of proposed QA process [Internet]. London: General Medical Council [cited 2020 Oct 19]. Available from: https://www.gmc-uk.org/education/standards-guidance-and-curricula/projects/review-of-our-quality-assurance-process/overview-of-proposed-qa-process [28]. GMC, General Medical Council; LCME, Liaison Committee on Medical Education; KIMEE, Korean Institute of Medical Education and Evaluation.

However, there have been recent changes in the accreditation process (Fig. 4). In the United Kingdom, the GMC is planning to apply a new quality assurance process starting in 2020 to ensure quality medical education [26]. The revised procedure includes a 4-year cycle declaration, a 1-year cycle self-assessment, triangulation, gap analysis, quality education, and regulatory assessment. Every 4 years, medical schools must meet the GMC’s standards or declare that they are working towards them. As the declaration entails signing a simple form, the GMC expects this change to lessen medical schools’ burden. However, if a school does not meet the criteria, the school’s declaration may be postponed. The US LCME certification cycle is 8 years. Every 8 years, accreditation is conducted via self-evaluation, visit evaluation, and an accreditation decision. Interim reviews are conducted annually by a questionnaire [27]. In Korea, the KIMEE grants certification periods of 2, 4, and 6 years according to the accreditation results. The KIMEE’s process consists of self-evaluation, visit evaluation, and an accreditation decision step for each cycle, similar to that in the United States. The interim evaluation consists of submitting an “interim evaluation research report” every 2 years, and visits for evaluation are not conducted.

Accreditation procedures are all composed of an interim evaluation and summative evaluation that determines accreditation, but the accreditation cycle, method, and procedure differ from country to country. In particular, the method of interim evaluation as a formative evaluation varies by country. The LCME and GMC monitor changes in medical schools with a questionnaire that is completed annually by medical schools, provide feedback on problems, and even visit medical schools directly if necessary [28]. In contrast, the KIMEE’s mid-term evaluation is based on medical schools’ reports regarding the accreditation criteria. There is no visit procedure, so the feedback process needs to be strengthened.

### The pedagogical value of medical school accreditation

Medical school accreditation is a part of education policy. Education policy is “the basic policy that the state and local governments publicly offer regarding education, which is an intentional and rational choice of the best alternatives for the purpose, means, and methods of education activities, and means to realize the purpose of education. At the same time, it refers to the education system and its operation” [29]. According to Wirt et al. [30], values are an essential factor in determining directions of educational policy. All policies, including education policies, go through a series of political processes to seek strategies for realizing specific values. Different values may conflict or complement each other, with some taking priority [31]. A policy may pursue a single value, but multiple values are usually combined. It is imperative to consider values because the nature and direction of policy may change depending on which value is given more importance [31]. Wirt et al. [30] presented 4 values to be pursued in educational policy: quality, efficiency, equity, and freedom of choice. Of note, equity means using public resources to reduce gaps between social classes and to distribute resources equally according to social norms [32]. Those 4 value concepts can serve as a useful framework for interpreting the educational value of medical school accreditation.

### Quality and equity

Accreditation can serve as a strong driving force to develop educational programs, including the curriculum. University officials who have experienced accreditation said that the actual necessity to reorganize the curriculum was due to accreditation, and the curriculum was reorganized according to the accreditation criteria [2]. This means that accreditation criteria are developing in a desirable direction to improve the quality of medical education by serving as useful and reliable tools, which is consistent with the achievement of the goals of accreditation.

However, accreditation may inhibit innovation in medical education. If genuine attempts to innovate education are judged not to meet accreditation standards, medical schools may be reluctant to innovate at all out of fear of undesirable accreditation results [2,20]. Ultimately, medical schools may be less active in providing excellent and innovative educational strategies, and their curricular reforms may be conservative if they aim only to meet accreditation standards. Therefore, it is highly likely that educational excellence, presented as a unique aspect of individual medical schools, will be reduced. This implies that accreditation standards can hinder the creative and innovative development of excellence in medical education [33]. Therefore, it is necessary to consider how the quality of medical education can be effectively improved while considering equity and excellence in medical school accreditation.

### Efficiency

The accreditation process can be a good opportunity for medical schools to restructure the curriculum, establish regulations, and monitor their educational systems [2]. It can enhance efficiency because accreditation mandates regulations and procedures for managing, supervising, and controlling educational activities. Nonetheless, economic efficiency may deteriorate because the medical school has to spend time and money every cycle as it undergoes accreditation [1]. If faculty members are required to work harder to meet the newly revised accreditation standards, they may experience “exhaustion of accreditation” [2]. The following is a complaint from a medical education officer who experienced difficulty communicating with the accreditation body during the accreditation process [34]:

“As the person in charge of medical education at my school, I used to feel that the KIMEE and I were on the same side, requesting improvement from the dean, the university headquarters, and the board of directors through accreditation. However, whenever I prepare for accreditation, I feel that each of the groups mentioned above is separately applying pressure on me. I no longer feel that the KIMEE is my ally.”
  Efficiency can be improved by revising and formalizing procedures.

### Choice

The value of choice in medical school accreditation is multi-faceted. From the student’s perspective, accreditation provides information about schools that they may apply to. For physicians, if their medical school meets international accreditation standards, it may broaden their career options [33]. From a national perspective, accreditation makes it easier to recruit doctors from other countries. However, international standards should not be regarded as promoting the brain-drain.

Nonetheless, the results of accreditation may harm schools’ reputation. Negative results can impact the quality of prospective students, which will eventually lead to a negative impact on university finances [2]. Accreditation can also potentially limit a medical school’s choices; schools often reform their curricula only based on accreditation standards, rather than making a variety of attempts to try different initiatives. In some countries, including China, there is a national curriculum, limiting each medical school’s choice of curriculum [35].

## Suggestions for actualizing the educational value of accreditation

### Efforts between the accreditation body and medical schools

Mutual trust must exist between the accreditation body and medical schools to increase the efficiency of the medical school accreditation process [3]. The necessary components to form an educational alliance between the 2 sides are as follows [15]:

- Mutual understanding of the purpose or goal of accreditation
- Shared views on the purpose of accreditation or the process of task accomplishment
- Beliefs based on mutual preference, trust, and respect

Medical schools trusting the accreditation body under a credible educational alliance will actively accept feedback from accreditation. A medical school’s attitude toward accreditation is largely influenced by a medical school’s culture and its members who transmit that culture. Therefore, each medical school’s “cultural construct” is an essential factor in determining changes related to accreditation. Culture is “a system of behaviors or lifestyles learned, shared, and transmitted by members of a specific group” and is “not behavior or an accident itself, but a standard or rule that gives meaning to it” [36].

The case of Yonsei University College of Medicine (YUCM) is an example of favorable feedback from the accreditation process. In the course of the curriculum development project, YUCM used accreditation results proactively. Although YUCM received the best score of the Korea University Education Council’s accreditation in 1996, the school immediately launched a curriculum development process after receiving the accreditation feedback. YUCM was told that the educational goals and objectives should be more concrete and be connected to the educational curriculum [37]. As a result, the educational objectives and goals were revised; a discipline-based curriculum was transformed into a system-based integrated curriculum. The KIMEE then actively embraced outcome-based and competency-based medical education and revised the accreditation standards. YUCM changed its curriculum into a student-centered, outcome-based, research-oriented integrated curriculum and implemented the first criterion-based pass/non-pass assessment system in Korea [38]. This change was possible because of “the cultural construct of YUCM placed a strong emphasis on education” [39].

The case of the Stony Brook University School of Medicine reflects the cultural conviction that the accreditation process requires participation from medical school administrators, leaders of various educational programs, many faculty members, managers of related institutions, and even students and residents [40]. This conviction encouraged student and faculty participation, allowing them to influence crucial institutional changes and flexibly respond to leadership changes. When preparing for the 2011 visit by the accreditation body, a leadership team was created to prepare even when the dean’s term was over. It was operated according to a 5-step process. As a result, a systematic and successful visit evaluation was possible.

### Regular review of standards and sharing of clear guidelines

While accreditation standards are the only way to unite the stakeholders of a medical school to understand the educational program’s values and the content of the curriculum [41], they also pose the risk of negatively impacting the reputation of specific medical schools that fail to meet the standards [33]. Therefore, constant review and appropriate revision of standards are essential to ensure reliability and validity. As an attempt to change according to new perspectives and reflections, an evaluation rubric can be used. A rubric offers different performance levels depending on how well the standards are met, so it is possible to keep track of universities’ progress according to the standards [13].

The WFME classifies accreditation standards at 2 different levels as an effort to improve accreditation. The first level corresponds to basic or minimum requirements, while the other level contains standards for improving quality [33]. The basic standards are expressed as “must,” indicating that all medical schools must meet these standards, while the standards for improving quality use the word “should.” Different standards are applied depending on the stage of a medical school’s development [13]. The Accreditation Standards of KIMEE 2019 (ASK 2019) are currently divided into basic and high-quality development standards. The current ASK 2019 decision guideline specifies that high-quality development standards are presented in the accreditation standards, but do not affect the accreditation results. These standards will improve the quality of medical education by simultaneously advancing the goals of equity and excellence. To improve the efficiency of medical school accreditation, a system that enables understanding and interpretation of standards to be shared between the accreditation body and the medical schools must be operated on an ongoing basis. The 2 sides should agree on accreditation standards and clear guidelines should be provided to help the medical school understand these standards accurately.

### Need for formative evaluation activities

We should not recognize medical school accreditation just as means to receive accreditation once every few years, but as a chance to build a system that can make improvements and advances in medical education. Regular evaluation is required to reach this goal [13]. Specifically, each medical school should conduct formative self-evaluation activities, which are a valuable way to review and recognize each medical school's unique educational process and philosophy [35].

For example, the US LCME regularly monitors medical schools through an annual questionnaire [40]. The annual questionnaire was developed by the American Medical Association and the American Association of Medical Colleges and includes content related to LCME accreditation standards, such as medical school size, number of faculty members, tuition, and finances.

The Council of Deans in the Association of Faculties of Medicine of Canada (AFMC) decided to mandate an interim review process (IRP) in addition to the 8-year term regular accreditation [40]. The IRP is an independent form of certification that can be considered as a kind of formative evaluation activity. The AFMC has developed a checklist for each element of the Committee for Accreditation of Canadian Medical Schools criteria, and each university conducts its evaluation according to the checklist.

Those intermediate evaluations may be able to minimize the exhaustion of certification. If medical schools share their evaluation experiences and the changes they make after accreditation, it will be possible to adopt the Canadian model. If the Korea Medical Education Association―a joint organization related to medical education―develops a model for formative evaluation that fits the situation in Korea, it may be helpful for the development of medical education in Korea.

## Conclusion

Improvements in the quality of medical education require voluntary reflection and criticism by representatives of medical schools, as well as evaluation and feedback from external organizations. This means that accreditation bodies and medical schools are on the same side as an educational alliance, with the common goal of improving the community’s health through high-quality medical education. When changing accreditation standards or processes, we should pursue values that match each medical school’s conditions. There is a need to help local medical schools to consider accreditation as an opportunity to achieve the development of medical education, rather than as an inevitable burden. We propose collaborating with medical schools, academic societies, and the KAMC with the accreditation body’s consent to provide an opportunity for formative evaluation, although it is not yet concrete. Rather than creating a new organization, it is necessary to actively discuss and study programs in which related institutions and universities actively participate as members of the educational alliance. Through these steps, medical schools across the country may be able to jointly develop and share a new culture in which accreditation is a real opportunity for development and change.

## Supplementary materials

Supplement 1. The audio recording of the abstract.

## References

[1] Gordon D, Karle H (2012). The state of medical and health care education: a review and commentary on the Lancet Commission report. World Med Health Policy.

[2] Blouin D, Tekian A, Kamin C, Harris IB (2018). The impact of accreditation on medical schools’ processes. Med Educ.

[3] Schirlo C, Heusser R (2010). Quality assurance of medical education: a case study from Switzerland. Neth J Med Educ.

[4] Maccarrick G, Kelly C, Conroy R (2010). Preparing for an institutional self review using the WFME standards: an international medical school case study. Med Teach.

[5] Tackett S, Zhang C, Nassery N, Caufield-Noll C, van Zanten M (2019). Describing the evidence base for accreditation in undergraduate medical education internationally: a scoping review. Acad Med.

[6] Abdalla ME (2014). Social accountability of medical schools: do accreditation standards help promote the concept?. J Case Stud Accredit Assess.

[7] Sethi A, Javaid A (2017). Accreditation system and standards for medical education in Pakistan: it’s time we raise the bar. Pak J Med Sci.

[8] Field MJ (2011). Medical school accreditation in Australia: issues involved in assessing major changes and new programs. J Educ Eval Health Prof.

[9] Zhang Q, Lee L, Gruppen LD, Ba D (2013). Medical education: changes and perspectives. Med Teach.

[10] Flexner A (1910). Medical education in the United States and Canada: a report to the Carnegie Foundation for the Advancement of Teaching [Internet]. http://archive.carnegiefoundation.org/publications/pdfs/elibrary/Carnegie_Flexner_Report.pdf.

[11] Irby DM, Cooke M, O’Brien BC (2010). Calls for reform of medical education by the Carnegie Foundation for the Advancement of Teaching: 1910 and 2010. Acad Med.

[12] Paul S, Lee JC (2012). The social transformation of American medicine.

[13] Abdalla ME (2012). Accreditation in medical education: concepts and practice.

[14] Telio S, Regehr G, Ajjawi R (2016). Feedback and the educational alliance: examining credibility judgements and their consequences. Med Educ.

[15] Telio S, Ajjawi R, Regehr G (2015). The “educational alliance” as a framework for reconceptualizing feedback in medical education. Acad Med.

[16] Van Zanten M, McKinley D, Durante Montiel I, Pijano CV (2012). Medical education accreditation in Mexico and the Philippines: impact on student outcomes. Med Educ.

[17] World Health Organization (2005). Accreditation of medical education institutions.

[18] Wall D, Swanwick T (2010). Evaluation: improving practice, influencing policy. Understanding medical education: evidence, theory and practice.

[19] Boelen C, Woollard B (2009). Social accountability and accreditation: a new frontier for educational institutions. Med Educ.

[20] Leinster S (2014). Role of accrediting bodies in providing education leadership in medical education. J Health Spec.

[21] World Health Organization, World Federation for Medical Education (2005). WHO/WFME guidelines for accreditation of basic medical education.

[22] Korean Institute of Medical Education and Evaluation (2016). KIMEE is recognized by the World Federation of School Education (WFME) as an accreditation agency [Internet]. http://kimee.or.kr/news-and-events/press-release/?pageid=2&mod=document&uid=189.

[23] Karle H (2006). Global standards and accreditation in medical education: a view from the WFME. Acad Med.

[24] The Executive Council (1998). International standards in medical education: assessment and accreditation of medical schools’ educational programmes: a WFME position paper. Med Educ.

[25] Grant J, Marshall J, Gary N (2003). Evaluation of the implementation in pilot sites of the World Federation for Medical Education’s international standards.

[26] General Medical Council (2019). Review of our quality assurance process [Internet]. https://www.gmc-uk.org/education/standards-guidance-and-curricula/projects/review-of-our-quality-assurance-process.

[27] Liaison Committee on Medical Education (2020). Guidelines for the planning and conduct of accreditation survey visits [Internet]. https://lcme.org/wp-content/uploads/filebase/guidelines_and_procedures/2020-Guidelines-for-Planning-and-Conduct_2020-04-09.docx.

[28] General Medical Council Overview of proposed QA process [Internet]. https://www.gmc-uk.org/education/standards-guidance-and-curricula/projects/review-of-our-quality-assurance-process/overview-of-proposed-qa-process.

[29] The Academy of Korean Studies (2019). Education policy [Internet]. https://encykorea.aks.ac.kr/Contents/SearchNavi?keyword=교육정책&ridx=0&tot=15.

[30] Wirt F, Mitchell D, Marshall C (1988). Culture and education policy: analyzing values in state policy systems. Educ Eval Policy Anal.

[31] Kim YI (2001). Educational policy and its intrinsic value. J Korean Educ.

[32] Jung HN (2016). A study on the typological classification of private tutoring market using the latent class analysis [dissertaion].

[33] World Federation for Medical Education (2015). Basic medical education: WFME global standards for quality improvement [Internet]. https://formacionenradiologia.files.wordpress.com/2018/09/wfme-2015-final-bme-global-standards.pdf.

[34] Jeon WT (2019). Accreditation in medical education.

[35] Geffen L, Cheng B, Field M, Zhao S, Walters T, Yang L (2014). Medical school accreditation in China: a Sino-Australian collaboration. Med Teach.

[36] Education Research Institute, Seoul National University (1994). The dictionary of educational studies.

[37] Committee on CDP2004 of Yonsei University College of Medicine (2002). Curriculum development 2004 report.

[38] Education Plan Committee of Yonsei University College of Medicine (2012). Curriculum development 2013 report.

[39] An S (2018). A diachronic study of the curriculum development of the undergraduate medical education: based on the case of Yonsei University Medical College [dissertation].

[40] Barzansky B, Hunt D, Moineau G, Ahn D, Lai CW, Humphrey H, Peterson L (2015). Continuous quality improvement in an accreditation system for undergraduate medical education: benefits and challenges. Med Teach.

[41] Lilley PM, Harden RM (2003). Standards and medical education. Med Teach.

